# Desmoplastic Fibroma of the Jaws: A Case Series and Review of Literature

**DOI:** 10.30699/ijp.2020.103833.2049

**Published:** 2020-01-26

**Authors:** Abbas Karimi, Samira Derakhshan, Monir Moradzadeh Khiavi, Farzaneh Mosavat, Faeze Mirjalili

**Affiliations:** 1 *Department of Oral and Maxillofacial Surgery* *, Dentistry School, Tehran University of Medical Sciences, Tehran, Iran*; 2 *Department of Oral and Maxillofacial Pathology* *, Dentistry School, Tehran University of Medical Sciences, Tehran, Iran*; 3 *Department of Oral and Maxillofacial Radiology, Dentistry School, Tehran University of Medical Sciences, Tehran, Iran*; 4 *Department of Oral and Maxillofacial Radiology, Dentistry School, Kashan University of Medical Sciences, Kashan, Iran*

**Keywords:** Benign, Desmoplastic, Fibroma, Jaw

## Abstract

Desmoplastic fibroma (DF) is a benign, locally aggressive neoplasm that rarely occurs in the facial skeleton. It usually presents during the first three decades of life. Due to its aggressiveness and high recurrence rate, early diagnosis is imperative, and complete surgical removal of the lesion is the treatment of choice. Herein, we present three cases of DF namely a 2 year-old girl with a mandibular DF, a 9 year-old boy with a maxillary lesion and a 1.5-year old boy with a mandibular DF. Complete clinicopathological information, treatment plan and long-term follow-up of patients are discussed. Histopathologic features of 3 cases revealed non-capsulated spindle cell tumor with fascicular or swirling patterns in incisional biopsy. Immunohistochemical staining was performed to make a definitive diagnosis. Strongly positive nuclear immunoreactivity for β-catenin confirmed the diagnosis of desmoplastic fibroma in 3 cases. Segmental mandibulectomy, partial maxillectomy and hemimandibulectomy were done for the cases. There was no recurrence in our reported cases after 8 and 11 months and 3 years follow up, respectively. It is noteworthy that despite the aggressive nature of DF, young patients often respond well to wide resection treatment.

## Introduction

Desmoplastic fibroma (DF) is a rare, benign, and locally aggressive, but non metastasizing, tumor with connective tissue origin (1,2,3). Similar to other intra-osseous fibrous lesions, it most often occurs in the metaphysis of the long bones. DF of the jaw was first reported by Griffith and Irby in 1965 (4). DF usually resembles a soft tissue desmoid tumor in terms of histological features. It clinically presents as a slow-growing, painless swelling. In the maxillofacial region, the posterior mandible is the most commonly involved site. Clinical and radiographic features often suggest the diagnosis of DF, but definite diagnosis can only be made by histopathological examination.

Herein, we report three cases of DF of the jaw and describe their surgical management and long-term follow-up results. A 2-year-old girl with an expansive mandibular lesion, a 9-year-old boy with a maxillary lesion and a 1.5-year old boy with a mandibular swelling with incisional biopsy reports of DF are presented. 

All parents of the patients signed informed consent forms.

## Case Report

**Case 1**

**Fig. 1:**
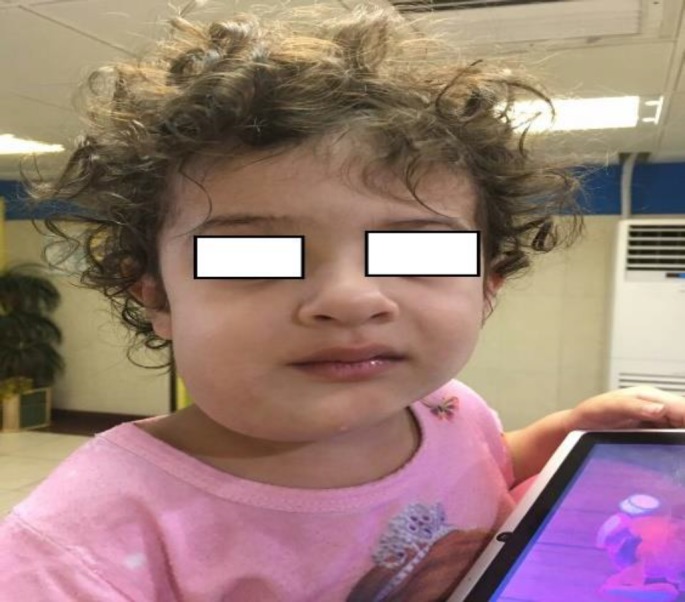
Clinical feature of patient shows swelling of the right mandible

A 2-year-old white girl was referred to an oral and maxillary surgeon with an abscess-like lesion in her right mandible from 2 months earlier **(**Figure 1). Clinical examination showed facial asymmetry due to a non-tender, firm, and prominent swelling in the right angle of the mandible. 

The overlying skin was normal and there was no history of trauma. The patient’s mother reported no change after 1 month of antibiotic therapy prescribed by a dentist on the first visit. 

**Fig. 2:**
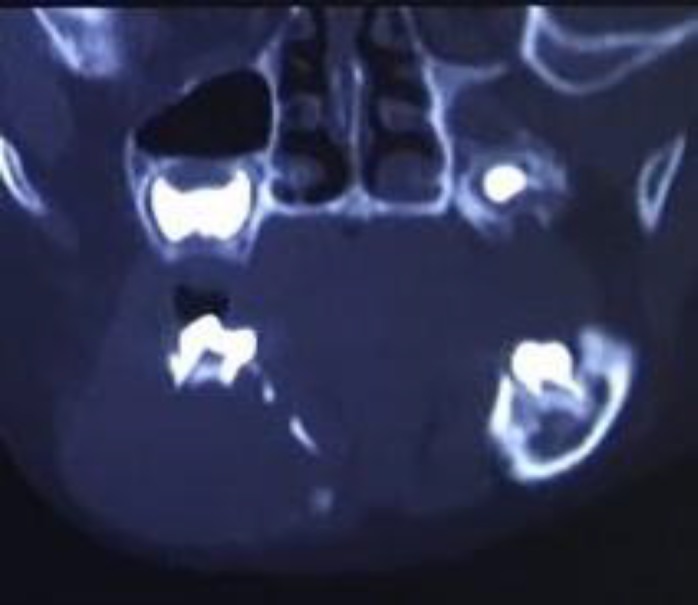
Coronal CT scan displays perforation of buccal and lingual cortices

Computed tomography (CT) scan of the lesion revealed a lytic lesion extending from the right angle of the mandible to the left lateral incisor, causing the expansion of both buccal and lingual cortical plates (Figure 2).

**Fig. 3:**
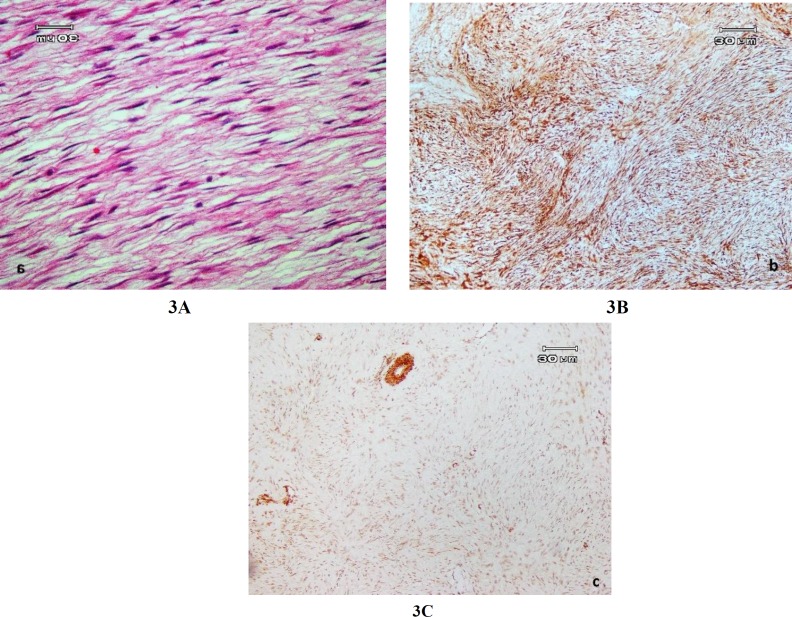
(a) Microscopic appearance of desmoplastic fibroma with bland spindle cells (hematoxylin and eosin, ×100); (b) Immunohistochemical staining. Note: the strong, diffuse positive staining of tumoral cells for β catenin antibody (×100) (c) Immunohistochemical staining: Mild diffuse positive staining of tumoral cells for α-SMA antibody. Note: severely positive staining of muscular vessel wall as internal positive control in the top middle of the figure (×100)

Incisional biopsy was performed and microscopic examination revealed a non-capsulated spindle cell tumor with short fascicular pattern in a collagenous stroma. There was no significant nuclear atypia, mitotic activity or necrosis (Figure 3A). These findings were diagnostically compatible with DF. Immunohistochemical (IHC) staining was performed to confirm the diagnosis of DF, which showed strongly positive nuclear immunoreactivity for β-catenin, which confirmed the definite diagnosis of DF **(**Figure 3B, C). 

The patient underwent an intra-oral surgical procedure by a sulcular approach for enucleation and curettage. Due to the loss of cortical integrity (noticed perioperatively), a segmental mandibulectomy through an extra-oral access with 1 cm safety margin was ultimately performed. Reconstruction of the mandible was performed using a 2.4-mm mandibular reconstruction plate (DePutySynthes, Switzerland) to restore the mandibular contour.

**Fig. 4:**
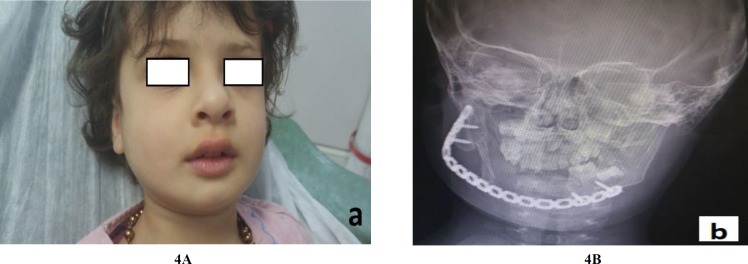
Clinical (A) and radiographic (B) features of case 1 at 6 months after the operation. No residual tumor is present

At the 8-month follow-up, there was no evidence of recurrence and it seemed that spontaneous regeneration of the anterior border of ramus had begun (Figure 4A & 4B).

**Case 2**

A 9 -year-old boy complaining of an acute swelling on the right side of his maxilla was referred to the Maxillofacial Surgery Department of Tehran University of Medical Sciences. Clinical examination revealed a firm expansion since 5 months earlier. The symptoms of pain, tenderness and numbness were not significant. The patient’s past medical history was unremarkable and he had no history of allergy. 

**Fig. 5:**
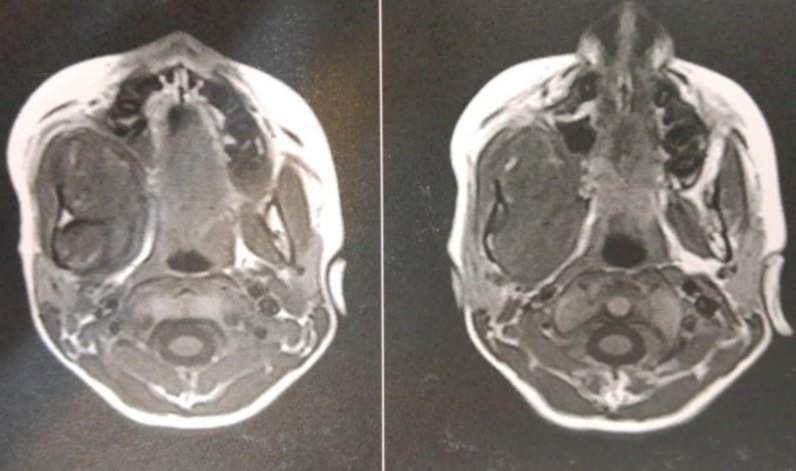
Preoperative MRI in axial plane showing the extension of the lesion

Magnetic resonance imaging (MRI) in coronal view revealed a large mass measuring 75 × 5 × 45 mm, involving the right maxilla and extending to the infraorbital area. The medial extension of the lesion had caused airway displacement and asymmetry as the result of changes in facial plane (Figure 5). 

**Fig. 6:**
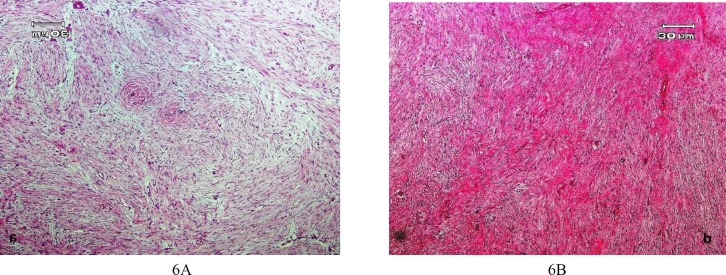
Microscopic appearance of desmoplastic fibroma. (A) spindle cell proliferation in a myxoid matrix. (B) Fibrotic matrix (hematoxylin and eosin, ×100)

The initial incisional biopsy revealed a tumoral mass composed of bland spindle cells proliferated in a fibrotic to myxoid matrix with a pattern-less arrangement (Figure 6A & 6B). 

IHC staining revealed positive immunoreactivity for CD 31 and SMA only in the vascular structure and CD 34 in some tumoral cells. B-catenin was strongly and diffusely positive in tumoral cells. Desmin and S-100 were negative in tumoral cells. IHC findings and histomorphometric assessments were compatible with juvenile DF. 

**Fig. 7:**
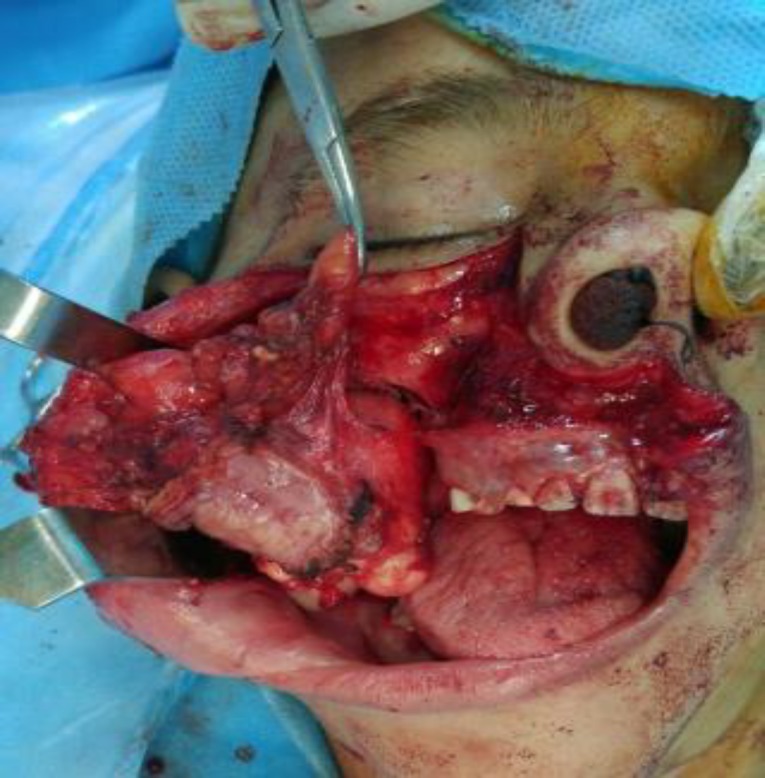
Intraoperative picture showing the lesion

Two weeks later, the patient underwent a surgical procedure via the Weber Ferguson approach. This approach is indicated for accessing the tumors involving the maxilla and extending superiorly to the infraorbital nerve and/or involving the orbit. It provides a wide access to all areas of the maxilla and orbital floor (Figure 7).

**Fig. 8:**
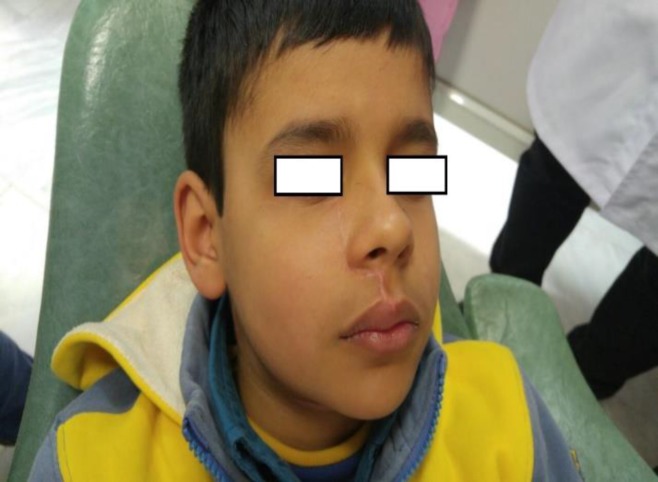
Clinical view of the case 2, 9 months after the operation

At the 11-month follow-up, the clinical and radiographic examinations showed no evidence of recurrence with acceptable esthetic and functional results (Figure 8).

**Case 3**

A 1.5-year-old boy with a swelling in the right side of his mandible was referred to an oral and maxillofacial surgeon. His parents reported a visit to a dentist and history of taking antibiotics due to a diagnosis of an odontogenic infection. The incisional biopsy of the unhealed lesion was compatible with benign spindle cell lesion. Extraoral examination revealed facial asymmetry due to the right mandibular swelling. There was no lymphadenopathy, paresthesia or pus discharge. His past medical history was unremarkable and the patient had no history of allergy. 

The surgeon requested to recheck the paraffin blocks to confirm the diagnosis prior to treatment planning. Histological examination during rechecking of the slides revealed tumoral tissue composed of proliferated spindle cells with ill-defined borders arranged in fascicular and swirling patterns in a non-collagenized stroma compatible with DF.

**Fig. 9:**
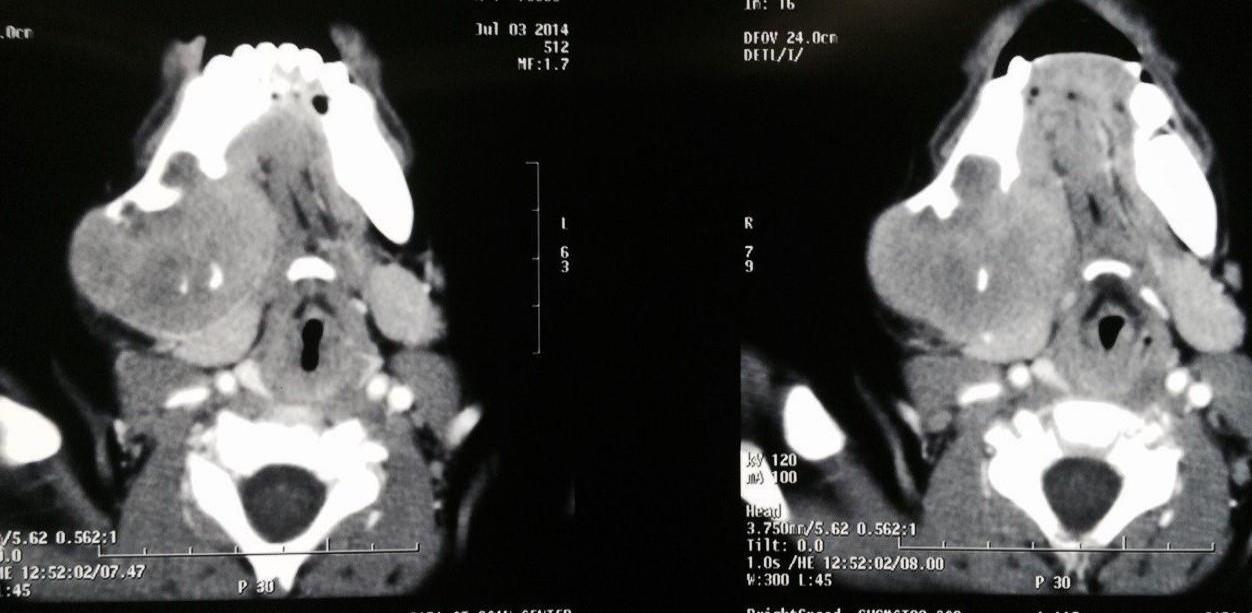
Axial CT scan of case 3 showing an expansile radiolucency in the right posterior mandible perforating the buccal and lingual cortices

Axial CT scan revealed a radiolucent, well-defined, expansile lesion in the right posterior mandible extending to the ramus area and perforating the buccal and lingual cortices. The lesion had extended to the hyoid bone in the medial aspect and had displaced the facial plane. The internal structure of the lesion appears to remnant bone. The lesion had not displaced the teeth (Figure 9). 

**Fig. 10:**
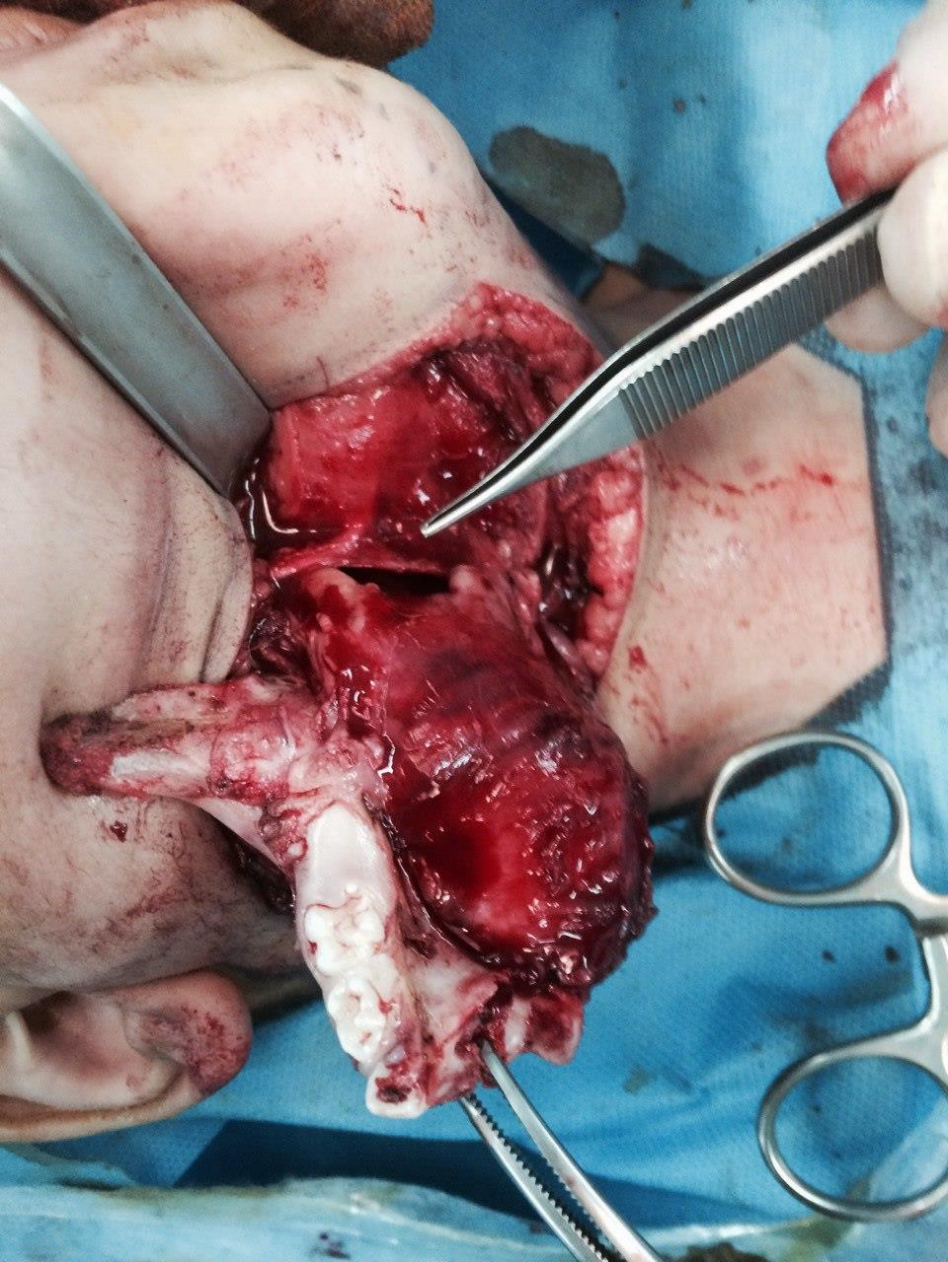
Trans-operative aspect of lesion resection in case 3

Two weeks after the biopsy and definite diagnosis of intraosseous fibromatosis (DF), the patient underwent right side hemimandibulectomy from the distal of the lateral incisor tooth to the ascending ramus of the mandible and coronoid process via an extraoral submandibular incision. The size of soft tissue mass was 6.5 x 5 x 3.5 cm (Figure 10).

At the 3-year follow-up, there was no evidence of recurrence with acceptable esthetic and functional results.

**Table 1:** Reported cases of desmoplastic fibroma of the jaws

Reference	Age		Gender	Site	Initial treatment	Follow up period
Saran RK* et al. *2000^13^	25		F	Maxilla	FNA, Excision	NA
Cupero* et al. *2001^14^	14		F	R posterior maxilla with orbital involvement	R maxillectomy wilh orbital preservation	2 y
Hereford* et al. *2001^15^	11		F	R mandibular angle	Marginal mandibulectomy	NA
Kaplan and Torske 2002^16^	3		M	R anterior mandible	Incisional biopsy, resection later?	NA
Vargas Gonzale* et al. *2004^17^	14		M	L maxilla facial region	Resection	NA
WippoldII* et al. *2005^18^	6 month		F	R mandible	NA	NA
Said- Al- Naief* et al. *2006^5^	8		M	R inferior border of mandible	Resection	4.5 years
Aurelio Lucchesi et al.2007^19^	11		M	L mandibular angle	Excision	33 month
Salah MB* et al. *2008^12^	20month			L anterior maxilla	?	?
Reid EN* et al. *2009^20^	60		F	Anterior mandible	Excision	NA
Schneider* et al. *2009^21^	23		M	L mandibrlar corpus	Excision	10 month
Summa* et al. *2010^22^	3		F	R mandible	Excision	Recurrence treated with chemotherapy
Boedker* et al. *2011^23^	44	F		L anterior zone of maxilla	Resection	NA
Mir. Mari* et al. *2011^24^	34	M		L anterior zone of maxilla	Resection	2 years
Ferri* et al. *2013^25^	3	F		R mandible	Partial mandibulectomy	36 month
	2	F		L mandible extending to cranial base	Partial mandibulectomy extending to cranial base	26 month
	2	F		R mandible	Resection	17 years
Oliveira Gondak* et al. *2013^26^	49	M		R maxilla	R maxillectomy	12 month
Gurrero et. al 2014^27^	70	F		L anterior mandible	Excision	5 years
Kalia* et al. *20152^8^	17	M		L mandibular angle	Excision	6 month
Woods* et al. *2015^7^	13	F		R mandible	NA	3.5 year
	57	F		L posterior mandible	NA	3 year
	20	F		L posterior mandible	NA	NA
Gersak* et al. *2016^29^	3.5	M		R mandible	Resection	NA
					
Reference	Age	Gender		Site	Treatment	Follow up period
Pereira* et al. *2016^30^	56	M		L mandible	Biopsy Resection later?	8 month
Nithya et.al. 2017^11^	35	F		L mandible	Excision	NA
Skinner et al 2014^2^	3.7	M		R mandible	R mandibulectomy	6 year
B. khatib, M.A. pogrel 2017^31^	8	F		L mandible	L mandibulectomy	14 year
	4	F		L posterior mandible	Resection	13 year
	2	M		L mandibular angle	Segmental resection	12 year

## Discussion

 DF of bone is comparable with soft tissue fibromatosis or intraosseous counterpart of soft tissue fibromatosis (2). The diagnosis of DF is challenging because of its slow onset and unremarkable findings (Table 1). The most common symptom of DF of the jaw is presence of a painless swelling that causes facial asymmetry (1, 2). All of our patients had slow-growing, painless lesions. 

DF usually occurs in the first 2 decades of life with a mean age of 16 years (75% < 30 years of age) with equal sex distribution. However, Said *et al.* reported a slight predilection for females in the jaw tumors (54 females: 45 males) (3, 5). Although the occurrence of DF in the maxilla has also been reported, mandible is the most common site of involvement in the head and neck and majority of the lesions are seen in the ramus, angle and posterior mandible (2,6). Femur (15%), pelvis (13%), radius (12%) and tibia (9%) are the other sites of involvement (7). 

According to the World Health Organization (WHO), DF is a benign tumor with low to variable cellularity; these cells can have ovoid to elongated nuclei with no polymorphism, atypia or mitotic activity (8).

The pathognomonic histological features of DF include the presence of mature fibrous connective tissue and spindle-shaped fibroblasts separated by abundant collagen fibers (3). Because of its pathognomonic histological features, it is important to distinguish it from other benign spindle cell tumors such as non-ossifying fibroma, nodular facititis, myofibroma, odontogenic fibroma and also fibrous dysplasia and low-grade fibrosarcoma. 

The histological features of fibrous dysplasia include hypocellular fibrous connective tissue with irregular shaped woven bone, giving it a ginger root appearance. Fibrous dysplasia can mimic DF especially in areas of dominant fibrous tissue with no osseous material. However, in fibrous dysplasia, the fibrous tissue is more hyper-cellular and vascular than DF.

Histopathological features of fibrosarcoma consist of low to moderate cellular proliferation of spindle cells, sometime without the classic feature of Herring bone. In contrast, there is no evidence of atypia, pleomorphism or mitotic figures in DF, which are the characteristic features of malignancy (2,7). Although there is no specific IHC marker for cells in DF, some IHC markers such as S-100, SMA, MSA, Ki-67, vimentin and B-catenin have been used to more accurately distinguish DF from similar entities. According to Woods *et al*., S-100 is negative in 93% of DF lesions, and 63% of lesions are negative for MSA or the HHF-35 (MSA antibody). Also, 92% positive immunoreactivity is seen for vimentin, 50% for B-catenin and 77% for SMA. Moreover, 100% of the lesions display less than 5% Ki-67 labeling, indicating a very low proliferation index (7). SMA and B-catenin were positive and S-100 was negative in one of our patients (case 2). 

The radiographic features of DF are nonspecific. It can be unilocular or multilocular with well-defined or ill-defined borders or irregular radiolucency. It usually has a lobulated appearance that resembles soap bubbles, and thus, it may mimic some other lesions of the jaws such as ameloblastoma, myxoma, aneurismal bone cyst, central hemangioma and eosinophilic granuloma. However, presence of coarse and irregular septa can help in its correct diagnosis (2,5). 

When the lesion damages the cortex, CT scan is recommended and if the tumor has extra-osseous growth, MRI is required to determine the exact extent of soft tissue invasion by the lesion (7,9). 

On T1-weighted MRI, the internal structure has a low signal which helps in determining the intraosseous extent because of the contrast with the high signal from the bone marrow (7). 

Several treatment modalities have been suggested for management of DF due to the aggressive nature of this benign lesion. However, because of its aggressive behavior and high recurrence rate, complete surgical resection of the lesion with safety margins is recommended. Curettage is usually sufficient for small mandibular lesions, but long-term follow-ups are necessary. The recurrence rate is about 40% to 47% in lesions treated by curettage or intralesional resection. Radiotherapy may serve as an alternative treatment when the lesion is inoperable but it is avoided in children because of postoperative complications (2,3,10,11). 

There was no evidence of recurrence of the lesion in our first and second cases after 6 and 9 months of follow-up, respectively. 

**Review of the Literature:**

To do a comprehensive literature review, a systematic search was conducted in the PubMed database, which revealed that only 96 cases of DF of the jaw have been reported from 1968 to 2017.

Said *et al.* reviewed the reported DF cases from 1965 to 2002. Thus, we decided to discuss the cases reported from 2000 until now (Table 1). There were only 30 patents with DF of the jaw during the designated time period, and we could not access the full text of the article reporting one of these cases (12). Of all, 60% of patents were female (17 females versus 12 males), which was similar to the results of Said et al (5). DF had occurred in a wide age range of patients; in this review, 20 patients (66%) were younger than 30 years of age. In 23 cases, the neoplasm was located in the mandible, mostly in the posterior region. No information was available about the initial treatment of 4 patients. Of all, 26% of the cases were managed by excision and the remaining 18 cases were treated by resection of the involved region with safety margins.

The follow-up period ranged from 6 months to 14 years; although, in 10 cases we had no information about the duration of their follow-up period and only in one patient the lesion had recurred and treated with chemotherapy (vinblastine and methotrexate). Thus, all patients were followed and there was no evidence of recurrence. 

## Conclusion

DF of the jaw is a rare, slow-growing and well-differentiated fibrous tumor with an aggressive potential for growth and recurrence. We reported three cases of DF of the jaws with no evidence of recurrence after several months. A wide local resection can be the best treatment option to minimize the recurrence rate of this benign aggressive tumor. 
